# Role of estrogen in hepatocellular carcinoma: is inflammation the key?

**DOI:** 10.1186/1479-5876-12-93

**Published:** 2014-04-08

**Authors:** Liang Shi, Yili Feng, Hui Lin, Rui Ma, Xiujun Cai

**Affiliations:** 1 Chawnshang Chang Live Cancer Center, Department of General Surgery, Sir Run-Run Shaw Hospital, Zhejiang University, Hangzhou 310016, China

**Keywords:** Estrogen, Estrogen receptor, Hepatocellular carcinoma, Inflammation

## Abstract

Hepatocellular carcinoma (HCC) is one of the most common malignancies worldwide and accounts for the third-leading cause of cancer-related deaths. Over the past decades, advances have been made in the field of surgery, but effective treatment of HCC is lacking. Due to a marked male predominance in morbidity and mortality in HCC patients, it has long been considered that sex hormones play a role in HCC development. Recently estrogen has been proven to exert protective effects against HCC through IL-6 restrictions, STAT3 inactivation and tumour-associated macrophage inhibition. While IL-6-dependent STAT3 activation is considered a key event in inflammation-induced liver cancer, the anti-inflammation effect of estrogen is well documented. The roles of the estrogen receptor and aromatase and interactions between microRNAs and estrogen in HCC have been investigated. In this review, we present a novel model to elucidate the mechanism of estrogen-mediated inhibition of HCC development through an anti-inflammation effect and provide new insights into the roles of estrogen in liver disease.

## Introduction

The treatment of liver cancer is a difficult task, especially among end-stage patients, whose lesions are usually thought to be unresectable. The development of HCC is considered the end result of most liver diseases, including viral hepatitis, cirrhosis and alcoholic liver disease. As a result of high-grade malignancy and lack of effectiveness of medical treatment, HCC is the third-leading cause of cancer deaths worldwide [1]. Furthermore, the incidence of HCC shows a regional divergence due to aetiology. In high-risk areas such as Southeast Asia and China, hepatitis B virus (HBV) infection, together with aflatoxin exposure, is the predominant risk factor. However, hepatitis C virus (HCV) infection has emerged as a more significant risk factor in Japan, North America and Europe. In these developed countries, the incidence of HCC is increasing [2,3].

Until now, only sorafenib is used as first-line therapy for patients with advanced HCC [4,5]. Sorafenib therapy for HCC has been proven to be safe and effective. Despite a statistically significant and clinically relevant improvement in median overall survival (OS) with sorafenib, some important questions of its application in advanced HCC remain unanswered [6]. For the better use of sorafenib in the clinical setting, the dosage, outcome prediction based on side effects and application of modified Response Evaluation Criteria in Solid Tumours (mRECIST) require more evaluation. Interested in obvious gender disparity in HCC occurrence [7,8], researchers have attempted to investigate the molecular mechanism underlying such disparity and developed effective therapy using sex hormones. Recently, work by Ma et al. showed an exciting potential of the combination of sorafenib and hormone-related therapy in HCC, which will be a better way to control advanced HCC [9].

Unlike breast and prostate cancer, which are modulated by estrogen and androgen, respectively, HCC may be modulated by both sex hormones during its initiation, progression and metastasis [8,10,11]. Elevated levels of androgen are considered to promote tumourigenesis, while studies in the past decades showed that the roles of estrogen in HCC are diverse, even opposite [8,12-14]. Fortunately, recent progress has shed some light on the precise mechanism of estrogen action in HCC. Although many aspects are still unknown, the anti-HCC activity of estrogen has been wildly accepted, and its protective effect might be related to its anti-inflammation effect. We thus assume that this anti-inflammation effect may provide a key to understanding the role of estrogen in HCC. More importantly, the anti-inflammation nature of estrogen may yield a new, promising approach to treat HCC. In this review, we focus on recent studies regarding the potential roles of estrogen in HCC.

### Early clinical trials of anti-estrogen therapy for HCC

At the very beginning of anti-estrogen therapy in medical practice, clinicians were inspired by the success of tamoxifen, which is a competitive antagonist of the estrogen receptor (ER), as a treatment for ER-positive breast cancer. In addition, estrogen was found to promote HCC in rats [15-19]. Some reports also showed that oral contraceptives (OCPs) led to a higher incidence of liver diseases such as focal nodular hyperplasia, liver haemangioma and hepatocellular adenoma, which are considered premalignant stages of HCC [20,21]. However, a systematic review concluded that no benefits could be gained by anti-estrogen in regards to overall survival and life quality [22]. And it is also suggested that OCPs might not to be a risk factor for HCC [23]. As a result, several hypotheses were proposed to explain the failures of using anti-estrogen in treating HCC, including the dysfunction induced by variant ER-α (vER-α) [24,25], regulation by the postreceptor signalling pathway and treatment with an insufficient therapy dose [26,27]. With further investigations, estrogen was found to suppress HCC [28], and this was supported by epidemiology data, which suggested an elevated incidence of HCC in postmenopausal female and suppression of such phenomenon by estrogen treatment [29,30]. Moreover, the prognosis of female HCC patients is much better than male patients [31]. These findings all imply that estrogen may act as a protective factor against HCC. Unfortunately, estrogen-related treatment is still impractical so far due to our limited knowledge [22].

### ER-α may be crucial to the estrogen-induced effect on HCC

The initiation of the canonical estrogen pathway depends on the binding of estrogen and its receptor. The ligand-bound ER then recognizes the estrogen response element (ERE) and regulates the transcription of target genes. Two subtypes of ERs have been found to date, ER-α and ER-β. Both ER subtypes are expressed in HCC and interact with each other [32]. Studies have shown that ER subtypes exert multiple functions in various stages of liver disease and participate in an extremely complicated signal transduction process. The different roles of the ER subtypes in liver disease, especially ER-β, have yet to be fully elucidated. Nevertheless, ER-α has been identified for a long time and has been extensively investigated [8].

**Figure 1:**
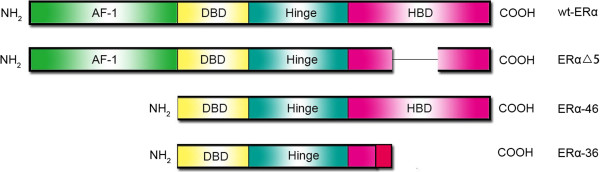
**Four types of ER-α variants in the liver.** Alternative splicing events generate several different isoforms of ER-α. In the liver, all types of ER-α have been reported and play a role in HCC. These variants are mainly AF-1- or HBD-depleted. ERα-36 has an additional 27-amino-acid sequence at its COOH terminus.

Wild type ER-α (wtER-α) contains 595 amino acid residues with a molecular mass of 66 kDa. It consists of a ligand-independent activation function domain (AF-1), a central DNA-binding domain (DBD) and a hormone-binding domain (HBD, also named ligand-dependent activation function domain, AF-2). The DBD and the HBD are linked by a hinge region [33]. Different ER-α splice variants, including ER-αΔ5 (incomplete HBD), ERα-46 (AF-1 deleted) and ERα-36 (AF-1 deleted and incomplete HBD), have been identified in the liver (Figure 1) [34,35]. The ER-α variants have been proven to be strong negative predictors for HCC [25,36,37]. ER-αΔ5 and hepatitis B virus X protein (HBx) can repress the transcriptional activity of wtER-α through an 17β-estradiol (E2)-independent method, and histone deacetylase-1 (HDAC-1) seems to be involved in the process [38].

A different expression pattern of vER-α can be observed in normal liver, cirrhosis and HCC tumour tissues (Table 1) [34], and a similar result can be obtained in liver cell lines. Furthermore, ER-α seems to lose function during liver disease progression, and dysfunctional ER-α could contribute to HCC development. In addition, ERα-36 negatively regulates the transcriptional activity of ER-66 and ER-β [39]. This crosstalk between ER subtypes forms a part of the complex ER signalling and has yet to be confirmed in HCC.

**Table 1:** The expression pattern of ER-α subtypes in different liver tissues

	**Normal liver**	**Cirrhosis**	**HCC**
**ERα-66**	High	Moderate	None
**ERα-46**	Moderate	Moderate	Moderate
**ERα-36**	None	Moderate	High

However, vER-α is considered a predictor of poor prognosis both in HCC and breast cancer [33]. In multiple-factor analysis, the ER-α type and bilirubin level were two independent risk factors for HCC [36]. The worst prognosis appears in patients with vER-α and hepatitis B surface antigen (HBS-Ag), and this may be explained by the suppression of transcriptional activity of the ER-α by HBx and ER-αΔ5. In addition, HBV infection could lead to genomic instability, which may contribute to the expression of vER-α [40]. Although tumour size was not obviously different in groups with different ERα types, vER-α-positive HCC presents a rapid growth rate and an increased ability of metastasis [36]. Therefore, vER-α-positive patients usually die of massive tumour invasion. In contrast, slow progressive liver failure due to cirrhosis kills wtER-α-positive HCC patients. Based on this finding, Villa et al. proposed that the treatment for wtER-α HCC patients should focus on chronic liver failure [36,41]. Furthermore, vER-α in chronic hepatitis and cirrhosis is also associated with high levels of oxidative stress-induced DNA damage and c-myc expression [42].

Recently, hypomethylation of the long interspersed nuclear element-1 (LINE-1) promoter was reported to correlate with poor outcomes of HCC [43]. Before this finding, LINE-1 hypomethylation was more frequently observed in hepatitis-related HCC to be accompanied by methylation of the ER promoter [44,45]. This hypermethylation of CpG islands in wtER genes may partially contribute to the significant downregulation of wtER and upregulation of vER-α in HCC [46]. The mechanism by which the expression pattern of the ER-α subtype is changed during liver tumourigenesis remains largely unknown. With more conclusive evidence, the expression pattern of the ER-α subtypes could serve as a potential prognostic indicator for HCC and provide a novel target for HCC treatment.

### Anti-inflammation effects of estrogen

The multitasking role of estrogen in inflammation is dependent on its concentration, cell type and context, and the anti-inflammation effect of estrogen has been further confirmed in many disease models, including liver disease. If B cell-dependent immunity or an overshooting fibrotic tissue repair process does not play a central role, estrogen would have a protective effect in chronic inflammatory diseases [47]. Some cytokines contribute to the occurrence of liver disease, and possible effects of estrogen on the release of proinflammatory cytokines *in vitro* have been discussed in a previous review (Table 2) [47].

**Table 2:** **Summary of possible effects of estrogen on cytokine production *in vitro***

**Cytokines**	**Effects of estrogen on cytokine production**	**References**
IL-1	Heterogeneous, depends on E2 concentration	[48,49]
IL-6	Mostly inhibition, promotion in synoviocytes (related to RA)	[48,50,51]
IL-8	Inhibition	[52,53]
TNF	Mostly inhibition	[48,54]
IFN-γ	Heterogeneous, depends on cell type	[54,55]
IL-4	Promotion	[55]
IL-10	Mostly promotion	[54,56]
TGF-β	Promotion	[57,58]

RA: rheumatoid arthritis.

Moreover, estrogen could inhibit the NF-κB pathway and block the expression of adhesion molecules. Inflammation factors, such as nitric oxide (NO) and reactive oxygen species (ROS) production are also downregulated in inflammation milieus. Given the emerging concept of cancer-related inflammation, it is of scientific and clinical interest to explore the possibility of using the anti-inflammation effect of estrogen in HCC prevention.

### IL-6 inhibition by estrogen

Hepatoma has been considered an inflammation-related cancer caused by chronic hepatitis [59-61]. During chronic inflammation, proinflammatory cytokines and immune cells create a tumour microenvironment that influences hepatocarcinogenesis. Among these, IL-6 is believed to be a key component in inflammation-associated tumourigenesis [62-64]. More importantly, estrogen has a significant impact on the production of IL-6. Preliminary studies showed that elevated IL-6 expression was associated with a high rate of metastasis and poor prognosis in HCC [63-65]. Concordant evidence from Naugler’s group showed that MyD88-dependent production of IL-6 contributed to gender disparity of HCC because IL-6 ablation protected male mice from HCC and that estrogen inhibited IL-6 production [51].

In the liver, the release of IL-6 from Kupffer cells (KCs) is modulated by MyD88-dependent NF-κB signaling, whose activation is triggered by IL-1α from dying hepatocytes [66-68]. According to the report by Naugler et al., E2 treatment could reduce diethylnitrosamine (DEN)-induced liver injury by inhibiting IL-6 production from KCs [51]. Moreover, E2 also provides protection in IL-6-treated mice, which suggests that E2 may inhibit downstream IL-6 signalling. The inhibition of the IL-6 promoter activity through inactivation of NF-κB and C/EBPβ causes downregulation of IL-6 [69]. These studies provide promising evidence for the higher incidence of HCC in males than females based on the role of IL-6 in anti-inflammatory effects [70]. However, a recent study on Forkhead box A (Foxa)-deficient mice showed that the IL-6 level did not correlate with tumour load when Foxa1/2 were only ablated in hepatocytes [71]. However, inflammatory monocytes that were recruited from circulation after HCC initiation exerted multiple effects on the tumour microenvironment. During HCC progression, tumour-associated macrophages (TAMs) replace KCs as the dominant modulator [61]. Taken together, these findings suggest that IL-6 is one of the regulators involved in the sexual dimorphism of HCC.

### Effects of estrogen on STAT3 activity

Signal transducer and activator of transcription-3 (STAT3) has been identified as a key regulator of macrophage functions and is involved in several programmes related to tumour progression [72-75]. Moreover, STAT3 signalling is a central signalling hub in cancer-related inflammation. The inflammatory microenvironment is orchestrated by numerous cytokines, chemokines and other mediators, and STAT3 is critical in regulating these inflammatory factors, such as IL-6, macrophage colony-stimulating factor, prostaglandin and cyclooxygenase-2 [76]. In STAT3-deficient mice, the liver tumour load caused by DEN treatment was significantly reduced [77]. In addition, the JAK/STAT3 pathway was enhanced in suppressor of cytokine signalling-3 (SOCS3) knockout mice, which were sensitive to hepatitis-induced hepatocarcinogenesis [78]. STAT3 signalling is negatively regulated through feedback loops. However, the polarization of macrophages to the M2 phenotype disturbs this homeostasis and keeps STAT3 activated; this, in turn, attenuates anti-tumour immune responses [79].

Recently, Hou et al. found that ER-α could suppress STAT3 activity in HCC cell lines and tumour tissues by elevating the expression of protein tyrosine phosphatase receptor type O (PTPRO) in female mice [80]. In E2-treated mice, an increased PTPRO level significantly inhibited HCC. Mechanistically, ER-α binds to EREs on the promoter of PTPRO then increases its expression. Moreover, it was found that the promoter region is methylated, which inactivates the PTPRO gene in HCC in human and rat models [81,82]. Methylation-mediated silencing of suppressor genes promotes carcinogenesis [83,84], but the methylation of *ptpro* that is modulated by ER-α must still be confirmed. In addition, this group found that PTPRO dephosphorylated STAT3 at Y705 and S727 then attenuated STAT3 signalling. Therefore, we could conclude that ER-α regulates STAT3 signalling by inhibiting IL-6 before STAT3 activation and directly suppressing STAT3 activity through PTPRO activation.

### Recruitment of ER-α depends on FOXA1/2

To exert multiple functions of estrogen, a ligand-bound ER must recognize ERE in target promoters. Previous studies have shown Foxa1/2 are involved in liver development and biological activity [85-88]. Additionally, in breast and prostate, the recruitment of ER-α and androgen receptor (AR) to target genes depends on FOXA1 [89-91]. No protective effect of estrogen could be observed in FOXA-deficient mice, and ER-α and AR exerted protective and oncogenic functions in HCC in a FOXA1/2-dependent manner [71]. According to this work, ER-α and AR are recruited to their target genes with assistance from FOXA1/2, and an ERE/ARE is found to be adjacent to FOXA binding sites on promoters. Moreover, an abundance of single nucleotide polymorphisms (SNPs) of the FOXA2 binding site is found on target genes during HCC progression in women due to attenuated affinity of FOXA2 and ER-α for their targets. In their study, estrogen was also found to enhance liver injury in mutant mice. In addition, genotoxic metabolites from estrogen contribute to carcinogenesis [92], hence, raising the notion that estrogen action in the liver is determined by the overall cellular context, rather than the hormone itself.

### Some microRNAs promote HCC through inhibiting ER-α

Previous studies found that miR-22 was downregulated in HCC and considered as a suppressor of cell proliferation [93]. However, Jiang et al. found that miR-22 was highly expressed in male HCC tumour adjacent tissue, and this expression was correlated with decreased ERα expression [66]. Furthermore, they showed that miR-22 inhibited ER-α transcription by directly targeting its 3′-UTR region, which was consistent with a previous study [94]. The deprivation of the anti-tumour effect of ER-α caused by miR-22 led to the carcinogenic process of adjacent liver tissues. Intriguingly, miR-18a, which has a high expression pattern in HCC tumour tissues, was also found to suppress the transcription of the ER-α gene [95]. However, miR-18a was not an inducer of female benign hepatoma in their research, which supported the notion that HCC and benign hepatoma are caused by distinct mechanisms. Actually, malignant transformation of OCPs-induced hepatic adenoma made estrogen as a HCC-promoting factor in early clinical trials [10].

Another study raised the possibility that miR-26a could prevent hepatoma cell growth through the repression of ER-α [96]. However, the marked decrease of ER-α and miR-26a in HCC tumour tissues indicated that downregulation of ER-α in HCC is mediated by a complex cellular network and not only by miR-26a.

### Role of ER-β in liver disease requires more investigation

ER-β shows strong anti-proliferative [97,98] and anti-inflammatory properties [99], and it is detected more frequently in patients with chronic liver disease than those with HCC [32], which implicates a protective role of ER-β in liver disease. Moreover, it has been shown that ER-β is overexpressed in HCV-related HCC tissues, but not in HBV-related tissues [100], suggesting that different mechanisms of HCC progression are induced by HCV and HBV. However, previous work showed the HBsAg could upregulate ER-β in *HBsAg* transgenic male mice, which raises the possibility that HBV infection may contribute to the gender disparity of HCC [101]. Intriguingly, ER-β displays anti-tumour effects in intrahepatic cholangiocarcinoma (IHCC) [102]. We believe that a better understanding of the roles of ER-β in liver disease will yield opportunities to develop novel therapies.

TAMs define an invasive microenvironment to promote tumour progression through multiple signalling pathways [103,104]. M2-polarised TAMs promote angiogenesis, metastasis and immune suppression by the secretion and modulation of cytokines, chemokines and growth factors [61]. A recent report revealed that the inhibition of the JAK/STAT6 pathway reduced TAMs polarization, thus suppressing HCC growth [105]. Such an effect is specifically caused by ER-β-induced SOCS1 expression. This finding indicates the protective role of estrogen through ER-β binding, but intriguingly, ER-β restrains the progression and metastasis of hepatoma [105]. In addition, unliganded ER-β regulates three classes of genes, whereas ER-α must be ligand-bound to regulate its target genes [106]. These findings provide novel insights into the role of ER-β in liver cancer. Unfortunately, the function of ER-β in HCC is largely unclear and requires further investigation.

### The synthesis of estrogen by aromatase in HCC

Elevated aromatase expression has been detected in hepatitis and HCC [107-109]. Intratumoural aromatase is considered a key inducer of estrogen-dependent neoplasm, such as breast, endometrial, and surface epithelial-stromal ovarian carcinomas [110]. The aromatase inhibitor fadrozole hydrochloride was demonstrated to counter spontaneous HCC in rats [111]. However, the contribution made by aromatase to the tumour microenvironment has not yet clarified. The elevated activity and expression of aromatase was detected in malignant human liver tissues and cells [108], and the influence of polymorphisms of the CYP19 (aromatase) promoter is associated with risk for HCC [112]. However, no conclusive evidence could be found to mechanistically link local estrogen production to HCC development. Consistent with the notion that estrogen prevents HCC by anti-inflammation effect, aromatase was found to be a risk factor only in non-viral hepatitis-related HCC.

## Conclusion

The effect of estrogen in HCC has turned from an oncogenic to protective role based on recent discoveries. Androgen promotes the development of liver and prostate cancer [113,114], whereas estrogen plays an oppositing role in the development of breast cancer and HCC [51,115,116]. How does such an antagonism exist? Pioneer work on this issue revealed that synthetic estrogen might promote liver carcinogenesis after DEN treatment in animal models [15-19]. Indeed, the precedence of estrogen or DEN treatment seems to have opposite effects on HCC development. Administration of estrogen prior to carcinogenic events such as DEN treatment, is believed to protect the liver from HCC [28], and this phenomenon is supported by a human model because estrogen usually functions in females before HCC initiation. Another possible mechanism for the oncogenic effect of estrogen is illustrated in the DEN-treatment model, where the NF-κB pathway is inhibited in hepatocytes (not in KCs). Blockage of NF-κB signalling in DEN-treated hepatocytes promotes carcinogenesis, because hepatocytes suffer severe cell death through necrosis and apoptosis [67]. In contrast, the inhibition of NF-κB signalling has a suppressive effect in hepatocytes of Mdr2^−/−^ mice, which is an inflammation-associated liver cancer model [68], consistent with the result in Huh7 cells [117]. Moreover, estrogen is found to attenuate HCC progression by regulating cell proliferation, invasion and apoptosis by inhibiting ER-α-induced NF-κB signalling [118]. NF-κB is highly associated with cancer-related inflammation, and estrogen inhibits NF-κB signalling; therefore, a novel model that fully captures the complex behavior of human HCC generation is required to understand the molecular mechanism by which the origin of HCC is modulated [119].

**Figure 2:**
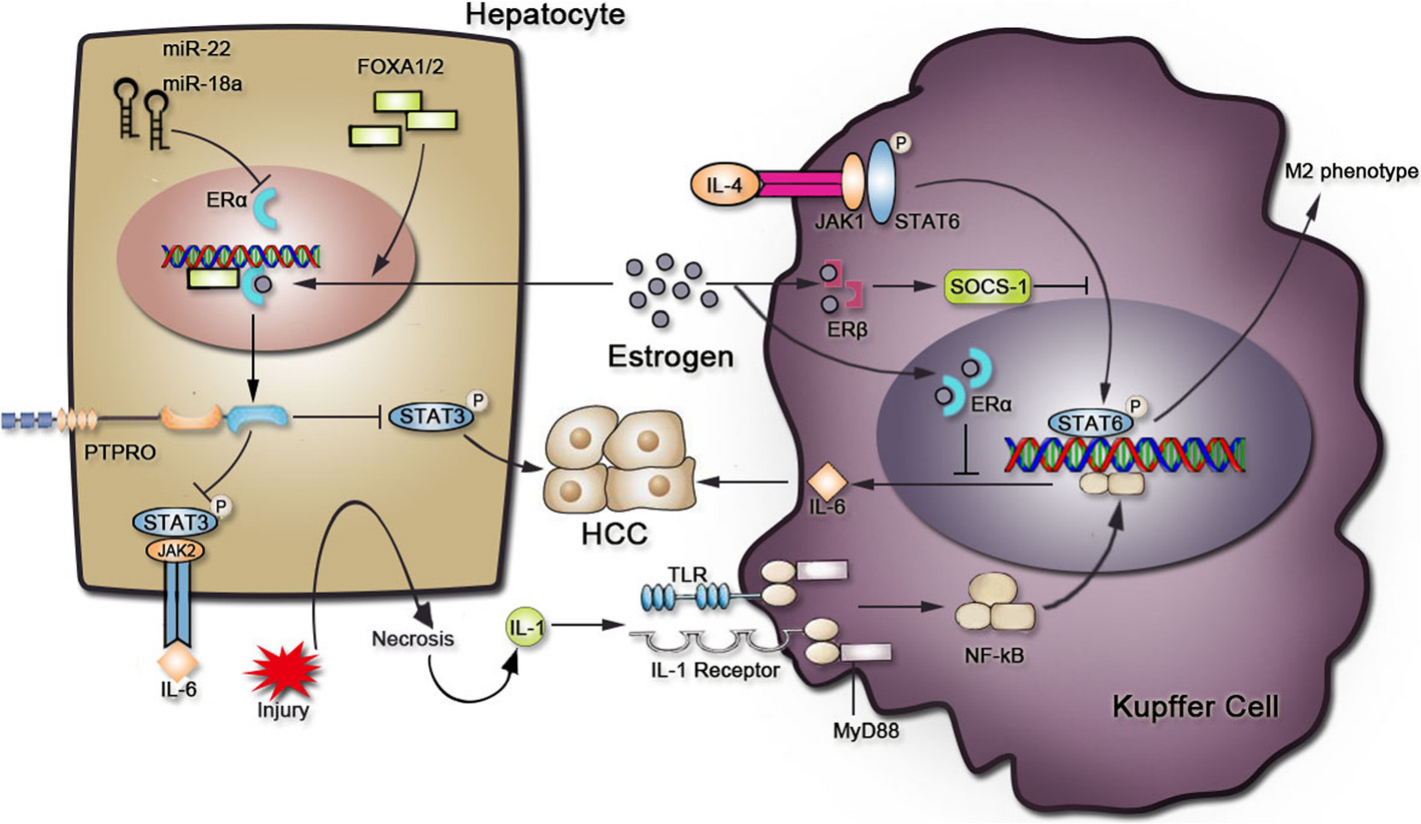
**Estrogen inhibits HCC development through its anti-inflammatory effects.** Estrogen exerts its protective effects through different pathways in KCs and hepatocytes. Ligand-bound ER-β inhibits M2 phenotype polarization in the ANA-1 cell line, and this procedure occurs mainly in TAMs, and not in KCs. Several miRNAs promote HCC by inhibiting ER-α. FOXA1/2 assists a ligand-bound ER-α to locate and bind to the ERE, and STAT3 plays a central role in these axes. Necrosis caused by liver injury defines the inflammatory microenvironment in the liver and eventually leads to HCC.

With emerging evidence supporting HCC as an inflammation-related cancer [59], we speculate that estrogen may, at least partially, play its protective role through its anti-inflammation effects. As described in detail in Figure 2, estrogen is involved in the regulation of the inflammation network in HCC by restraining of proinflammatory cytokines and inhibiting downstream signalling pathways. However, it is also reported that estrogen promotes hepatocytes proliferation [120]. Here, we believe that estrogen exerts promoting and inhibiting effects on HCC development, but, in tumour milieus, it is generally accepted as a mediator of anti-inflammation. The oncogenic effect of estrogen could also play a part in tumourigenesis, as cancer cells will use all of the help they can get. That is, both faces of estrogen are retained in HCC, but it protects females from HCC because inflammation is the key event for HCC development. The cellular milieus help estrogen protect from liver cancer. However, evidence for the anti-inflammatory effect of estrogen in HCC is limited; and uncovering how estrogen protects from HCC development would provide novel therapeutic approaches in drug design and cancer therapy (Table 3).

**Table 3:** Inhibition of inflammation factors by estrogen in HCC

**Inflammation factors**	**Models**	**References**
IL-6	DEN-treated mice	[51]
STAT3	DEN-treated mice, Huh-7 cells, SMCC-7721 cells	[51,80]
NF-κB	DEN-treated mice, HepG2 cells, H22 cells	[51,118,121]
TAMs	Orthotopically and ectopically implanted HCC mice, coculture system of macrophages and Hepa1-6 cells	[105]

It is important to bear in mind; however, that estrogen may cause some adverse effects in patients, especially in males, who form the majority of HCC cases. Therefore, the method for using hormone-related therapy to treat HCC requires rigorous testing and validation. In the future, finding the exact point at which estrogen switches its role from oncogenic to suppressive in HCC will enable us to establish a model to mimic chronic inflammation during HCC development.

## Abbreviations

HCC: Hepatocellular carcinoma; ER: Estrogen receptor; OCPs: Oral contraceptives; ERE: Estrogen response element; wtER-α: Wild type ER-α; vER-α: Variant ER-α; E2: Estradiol; HBx: Hepatitis B virus X protein; HBS-Ag: Hepatitis B surface antigen; LINE-1: Long interspersed nuclear element-1; KCs: Kupffer cells; DEN: Diethylnitrosamine; Foxa: Forkhead box A; TAMs: Tumour-associated macrophages; STAT: Signal transducer and activator of transcription; SOCS: Suppressor of cytokine signalling; PTPRO: protein tyrosine phosphatase receptor type O.

## Competing interests

The authors declare that they have no competing interests.

## Authors’ contributions

LS performed article searches, drafted the manuscript and figures and revised the manuscript. YF performed language correction and contributed to refinement of the manuscript. HL and RM participated in information updating and viewpoint complementarity. XC provided the original idea, administrative support and financial support. All authors read and approved the final manuscript.
